# Assessment of addiction behavior and spermatogenesis in glial cell line-derived neurotrophic factor-treated cannabis-addicted rats: An experimental study

**DOI:** 10.18502/ijrm.v23i2.18487

**Published:** 2025-05-01

**Authors:** Rozhina Laleh, Mitra Heydari Nasrabadi, Parvin Khodarahmi, Jamshid Soltani

**Affiliations:** ^1^Department of Biology, Parand Branch, Islamic Azad University, Tehran, Iran.; ^2^Department of Medical Radiation Engineering, Parand Branch, Islamic Azad University, Tehran, Iran.

**Keywords:** Wistar rat, Cannabis smoking, GDNF growth factor, Spermatozoa, Histological techniques.

## Abstract

**Background:**

Cannabis addiction poses risks to male fertility by lowering levels of glial cell line-derived neurotrophic factor (GDNF), which is vital for spermatogenesis.

**Objective:**

This research aimed to determine if the injection of exogenous GDNF into the brain of cannabis-addicted rats has a positive impact on their behavior and spermatogenesis.

**Materials and Methods:**

This study involved 15 male Wistar rats divided into 3 equal groups: control, model, and experimental. Cannabis addiction was induced in the model and experimental groups using a smoking machine with a 0.25 gr dose per 5 rats, and the experimental group received a 0.5 mg GDNF treatment via stereotaxic injection. Behavioral changes were assessed through plus maze, open field, and sucrose preference tests before and after treatments. Sperm parameters were evaluated with H&E staining, sperm morphology with Diff-Quik staining, DNA damage and viability with acridine orange and trypan blue staining.

**Results:**

Addicted rats displayed increased anxiety, which was improved by GDNF treatment (p < 0.05). Although cannabis significantly reduced germ cells and the size of the testis and epididymis compared to controls (p = 0.0006, p = 0.003), GDNF had a limited effect on these aspects. Cannabis significantly altered sperm morphology (p = 0.0016), but GDNF reversed abnormal sperms. GDNF improves sperm quality, reverses cannabis-induced sperm grading alterations (grade C, p = 0.0295), reduces DNA damage significantly (p = 0.0242), and enhances sperm viability, highlighting its potential to counteract some of cannabis's harmful effects on male reproductive health.

**Conclusion:**

The findings of this experiment suggest that exogenous GDNF could be a potential therapeutic agent for cannabis addiction and sperm parameters.

## 1. Introduction

Cannabis contains over 500 compounds, including 104 identified cannabinoids, which interact with cannabinoid receptors and can disrupt the endocannabinoid system, potentially leading to infertility (1). The endocannabinoid system components are present in semen plasma, male reproductive organs, and germ cells, impacting reproductive processes (2). Prolonged marijuana use may further impair fertility by altering testosterone levels and sperm motility (3).

Spermatogenesis depends on a supportive microenvironment within the testis, largely maintained by Sertoli and Leydig cells. Cannabinoid receptors on these cells play a key role in chemical balance, influencing reproductive processes (4).

Recent studies reveal that cannabinoids, primarily Δ 9-tetrahydrocannabinol and cannabidiol, interact with CB1 and CB2 receptors present on Sertoli and Leydig cells, which are crucial for maintaining the testicular environment necessary for healthy spermatogenesis (5).

Experiments to study the biology of addiction, have focused on the mechanisms through which drugs of abuse, drive changes in the functioning of neurons and neural circuits. Glia have often been ignored in these studies. There were significant reductions in the mRNA levels of neurotrophic factors (brain-derived neurotrophic factor, glial cell line-derived neurotrophic factor [GDNF], and neurotrophin nerve growth factor) and their receptors following morphine dependence. The expression of almost all neurotrophic factors increased after morphine withdrawal (6). GDNF is a type of brain growth factor typically present in all parts of the central nervous system during development. Still, in the adult brain, its expression is limited to certain areas and is used to create differentiation (7). Studies have shown that GDNF is abundantly produced in the NA in the adult brain and transported retrogradely to the ventral tegmentum (VTA), where it binds to GDNF family receptor α1 and activates its signaling pathway. Drug abuse in the brain is primarily linked to the mesolimbic dopamine system, which involves the branching of VTA to nucleus accumbens (8).

Recent findings show that GDNF may have a regulatory role in the actions of abused drugs (9). Most studies found that activation of the GDNF pathway reduces biochemical and behavioral changes in rats exposed to drugs (10).

This study was conducted considering previous studies and the importance of the impact of cannabis addiction, following the increasing use of cannabis among young people. The therapeutic effects of GDNF on cannabis treatment were studied to understand their impact on the future of male reproduction. The objective of this study was to investigate how GDNF affects the behavioral performance and spermatogenesis of rats addicted to cannabis. If GDNF treatment shows efficacy in improving addiction behavior and reversing damage to spermatogenesis, it could serve as a promising therapeutic strategy for people with cannabis addiction. This dual therapeutic impact -addressing both addiction and its reproductive consequences- could be groundbreaking in addiction medicine.

## 2. Materials and Methods

### Animals and drug treatments

#### *Cannabis sativa* (*C. sativa*) addiction in rats of model group 

This experimental study was conducted from December 2022 to January 2023 in the animal house of Parand Branch, Islamic Azad University, Tehran, Iran. Also, from January 2023 to February 2023, behavioral and histological studies were performed in the specialized laboratories of HistoGenotech Co. A total of 15 male Wistar rats (180 gr, 4 wk) were acquired from Pasteur Institute of Iran. Rats were kept at an ambient temperature between 20–22 C, which was controlled and ventilated by a split air conditioner, and the light cycle of 12 hr light/dark cycle were applied (11). The rats were randomly assigned to 3 experimental groups: a group of rats addicted to cannabis (n = 5), a control group (n = 5), and a group of cannabis-addicted rats treated with GDNF (n = 5). The sample size in this study was calculated using G power software. *C. sativa* was obtained from the Faculty of Pharmacy at Tehran University of Medical Sciences, Tehran, Iran with the herbarium code TEH-7151. The sample was duly authenticated by an expert from the Faculty of Pharmacy. The *C. sativa* preparation was conducted using 0.25 gr rolls. Addiction induction sessions were undertaken twice a week, with 6 sessions. Each session involved administering 0.25 gr of substance per 5 rats. The mechanical engineer created and manufactured a smoking machine with a voltage of 12V for this purpose (12).

#### Stereotactic surgery for GDNF injection in rats of experimental group

First, the rats were anesthetized with ketamine (100 mg/kg) and xylazine (10 mg/kg) intraperitoneally. Then animals were positioned in the stereotaxic apparatus (ST3000, SanatAzma, Iran). The surgical procedure commenced by disinfecting the incision site on the skull. A sterile surgical blade was then employed to create an incision along the middle sagittal groove of the skull, spanning the distance between the eyes and ears. The Bregma point corresponds to the intersection of the coronal suture and the sagittal suture, where Lambda and its intermediate line were accurately identified. The Paxinos Watson rat brain atlas was utilized to determine the specific features of the hippocampus (13). Subsequently, an injection of GDNF (MERC, GERMANY) was administered within the hippocampal region, employing the following co-ordinates: mediolateral ± 2.4 mm, anteroposterior -3.8 mm, dorsoventral +2.7 mm, and BREGMA -3.10 mm. Following the process of site drilling, a precise quantity of 0.25 μg of GDNF was meticulously administered into both the right and left hippocampal regions through a Hamilton syringe (Hamilton, U.S.A.) (14). The surgical wound was closed using sutures and treated with gentamicin to prevent wound infections.

Samples were collected to investigate the effect of GDNF injection on the spermatogenesis process, one week after the recovery (15, 16).

### Behavioral assessment

To evaluate the behavior of rats, 3 behavioral tests, including the plus maze test, open-field test, and sucrose preference test (SPT), were considered. The plus maze test was utilized to measure anxiety behaviors and verify the general motor activity of rats (17).

The maze consisted of 2 open arms of 50 × 10 cm, and 2 closed arms of the same size with 40 cm walls and an open ceiling at a height of 50 cm from the floor. The open-field test is employed to assess both behavioral responses and anxiety. An open box was used to perform this test, the bottom of which was divided into several equal squares by lines. The width of this box was divided into 2 areas: the perimeter and the center. The rat movement to the central area of a box with dimensions of (44 cm wide × 44 cm long × 40 cm high) was checked for 10 min and its location was tracked, recorded, and analyzed by a digital camera installed on top of the box. Anxiety behavior was determined by the time spent in the central zone (18). During all experiments, the open field box was cleaned with 30% alcohol before placing the following rat. Both tests evaluated behaviors such as crossing, risky behaviors, searching, grooming, and sniffing. The control, addicted, and GDNF-treated (experimental) rats had their behaviors counted and compared over a period of 5 min. A SPT was performed to evaluate depression in rats. To perform this test, 2 bottles (the capacity of each container is 700 ml) were used, one containing water and the other containing 2% sucrose. In order to reduce the stress response to new conditions, 24 hr before the test, the rats were allowed to freely choose one of the 2 bottles to drink for 3 hr. After becoming accustomed to the environment, the test lasted for 4 days, with each day consisting of 8 hr. The bottles were weighed at the beginning and end of each 8-hr period in order to calculate the amount consumed from them, and at the end of the 4 ${ߐ}^{\text{th}}$ day, the ratio of sucrose to the total volume consumed was measured using the following formula: [sucrose intake/(water intake + sucrose intake)] × 100 (19).

### Histologic study

For histological evaluations, the rats of all 3 groups were euthanized using CO_2_, and their testicles and epididymis were removed. The dimensions of both testes and epididymis were measured with a caliper, and their weight was measured with a digital scale with an accuracy of 0.01. The samples were then placed in Boen's fixative. The slides were examined with a light microscope after being stained with hematoxylin-eosin, and transverse sections with a thickness of 5 μm were made. The spermatogenic tubes and germinal layer were measured using a microscope. The number of germ cells, spermatogonia, primary spermatocytes, spermatids, and sperms were measured on every slide. After separating the epididymis, they were cut into small pieces in 5 ml of Ham's F10 solution and placed in an incubator at 37 C with 5% CO_2_ for 5 min until the sperms were released from the epididymal tubules. A drop of the resulting suspension was placed onto the slide to count the sperm cells. The number of sperm cells per milliliter was determined by multiplying the result by 50,000 (20).

### Sperm parameters

Sperm morphology was analyzed by using Diff-Quik staining (21). After placing 10 μl of the sample on the slide, the second slide was taken at an angle of 45 C to the first slide and moved in just 1 sec. The slide was fixed and stained after it was prepared, smeared, and dried in the vicinity of the air. Diff-Quik staining was employed for this task, causing the acrosome area of the sperm head to appear pale blue and the back area of the acrosome to appear dark blue. The middle part has a hint of red, and the tail area is either blue or red. The kit (Faradid pardaz pars, Iran) contains staining solution I (eosinophilic xanthines) and solution II (basophilic thiazine). In addition, 95% methanol was utilized as a fixative. The slides were first immersed in 95% methanol for an hour, then subjected to staining solution I for 10 sec and solution II for 10 sec. They were rinsed with tap water 10 times. At last, the slides were analyzed using a microscope.

### DNA fragmentation

Acridine orange staining was used to evaluate the normality of sperm DNA (22). Sperm samples isolated from the epididymal tail were washed 3 times in 0.1 M phosphate-buffered saline (Merck, Germany) at room temperature. After centrifugation, the sperm sample was resuspended again, and approximately 10^6^/ml and 15 μl of sperm suspension were smeared on a glass slide. After drying, the sperm slides were put into Carnoy's solution (methanol/acetic acid ratio 3:1) and kept there for 3 hr. They were subjected to an Acridine orange (Merck, Germany) staining solution for 5 min. For this purpose, 10 ml of 1% Acridine orange (AO) solution in distilled water was added to a mixture of 40 ml of 0.1 M citric acid and 2.5 ml of Na_2_HPO_4_.7H_2_O with a concentration of 0.3 M (pH 5.2). 1% AO stock solution can be stored in the dark at 4 C for 4 wk. Next, the slides were gently rinsed with distilled water, and the percentage of sperm with normal DNA was determined. The fluorescence microscope (Olympus Co., Japan) was used at × 400 magnification with a 450–490 nm wavelength. Sperms with normal and double-stranded DNA were observed with green fluorescence, while sperms with denatured DNA were observed with red color (23). Epididymal sperm analysis was performed according to the World Health Organization guidelines.

### Sperm viability 

Trypan blue staining was employed to examine the viability of the sperm (24). After washing with phosphate-buffered saline (Merc, Germany), sperm samples were mixed with 4% trypan blue (Merck, Germany) at a ratio of 1:1. The compound was placed under the Neubauer slide with a drop of 10 μl. The sperm were photographed or counted in the shortest possible time, within 1–2 min. Cell death and a decrease in living cells occur with increased time.

**Figure 1:**
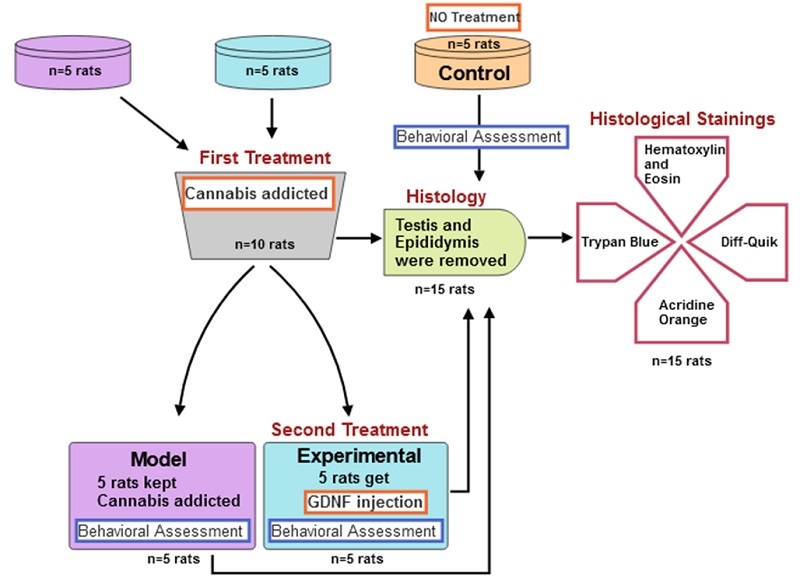
Flowchart of material and methods.

The percentage of sperm survival is determined by the ratio of live cells to total counted cells (25) (Figure 1).

### Ethical Considerations

The study and all procedures were performed according to the principles of the Declaration of Helsinki. The present research has the code of ethics from the Ethics Committee of Varamin Pishva Branch, Islamic Azad University, Tehran, Iran (Code: IR.IAU.VARAMIN.REC.1401.048).

### Statistical Analysis

In this research, data were divided into 2 categories, sperm staining and behavioral studies, in 3 groups: control, model, and experiment. The results were expressed as the mean ± SD.

The statistical analysis for quantitative variables and the mean differences between all groups were compared using one-way analysis of variance (ANOVA), followed by Tukey's post hoc to check the existence of significant differences between these 3 groups. P < 0.05 was considered significant in all stages.

SPT was calculated using the following formula: [sucrose intake / (water intake + sucrose intake)] × 100.

GraphPad Prism v8.0.2 software (San Diego, CA, USA) for the stainings and SPSS software for Windows version 26.0 (SPSS Inc., Chicago, IL, USA) was used to analyze the data of behavioral assessments.

## 3. Results

### Behavioral assessment

The plus maze and open-field tests, revealed that cannabis-addicted rats experienced a significant decrease in crossing and rearing behavior, but it increased again after GDNF treatment. Grooming and sniffing movements were significantly lower in cannabis-addicted rats than in control rats. These 2 movements were significantly less in rats treated with GDNF compared to those addicted to cannabis. The napping movement was observed only in the rats of the cannabis-addicted group, and the defecating movement was observed only in the control group rats. In the plus maze test, all 3 groups had similar risk behaviors and no significant differences were observed. In contrast, in the open-field group, risk behavior was significantly reduced in cannabis-addicted rats. The percentage of sucrose preference in the control group was 79.33%, but it decreased significantly and reached 42% in the rats addicted to cannabis. The percentage of sucrose preference in rats treated with GDNF was calculated at 70%, which was not significantly different from the control group (Table I).

### Histologic study

**Figure 2:**
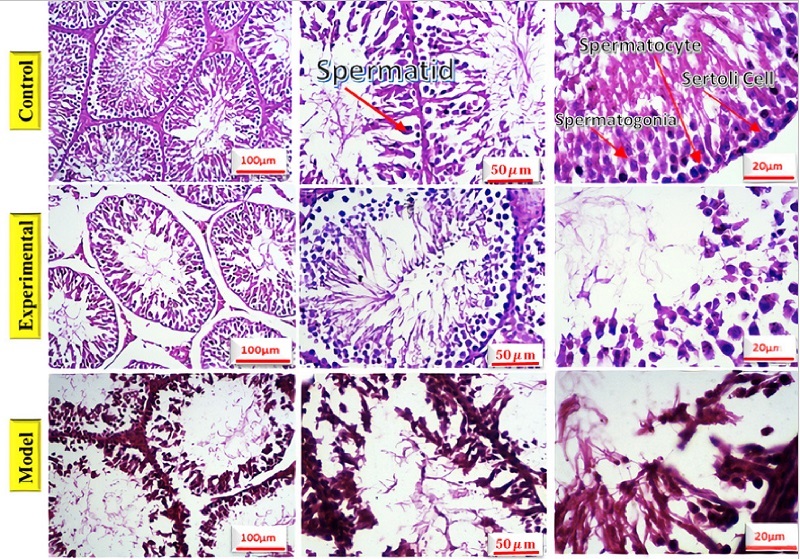
Evaluation of testicular tissue and epididymis in healthy control rats (control), cannabis-addicted rats (experimental), and GDNF-treated rats (model).

The testicles and epididymis tissue of rats were examined using hematoxylin-eosin staining, in figure 2, different sections are shown with various magnifications. Table II depicts the number of spermatogonia, spermatocytes, spermatids, Sertoli cells, and Leydig cells, the thickness of the germ layer, the thickness of the basement membrane, and the thickness of the outer diameter of the seminiferous that are compared in 3 groups. No significant change was observed in the rats treated with GDNF compared to the addicted rats. The thickness of the internal diameter of the seminiferous tubule in cannabis-addicted rats was significantly more than in the control group. The internal diameter of the seminiferous tubule was not significantly different in GDNF-treated rats compared to cannabis-addicted rats. It appears that GDNF has little effect on the improvement of testicular and epididymal tissue in cannabis-addicted rats.

### Sperm parameters

Sperm motility was assessed using a grading system. Compared to the control group, the results showed a decrease in sperm motility in grades A and B, and an increase in grade D in cannabis-addicted rats (Table III). GDNF treatment increased the motility of grades A and B. However, GDNF did not have much effect on grade D. Cannabis-addicted rats showed a significant increase in grade C compared to control rats. In contrast, GDNF was found to reduce grade C compared to addicted rats.

Detailed analysis of semen classifies sperm motility into one of the following grades:

• Grade A, they are fast-moving sperm that swim rapidly in a straight direction.

• Grade B, they are slow-moving sperm that move forward but in a random line.

• Grade C, are non-progressive sperm that can move their tails but cannot move forward.

• Grade D, these sperm are immobile and do not move at all.

Sperm counts showed that their number was significantly reduced in cannabis-addicted rats compared to control rats. GDNF treatment resulted in a slight increase in sperm count (Table III). According to these findings, GDNF treatment was able to bring sperm motility parameters closer to those of healthy groups and eliminate the adverse effects of cannabis.

### Morphology of sperms

**Figure 3:**
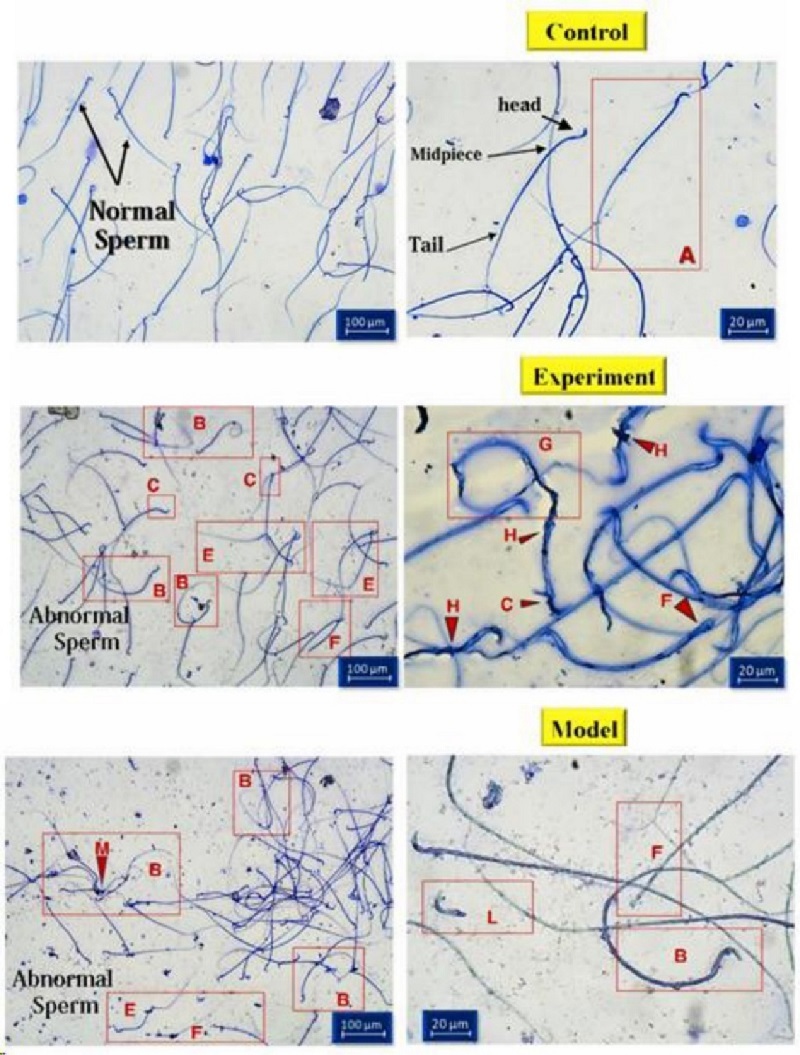
Sperm morphology assessment using Diff-Quik staining: A) Normal sperms of control rats with heads, midpieces, and tails (arrows) B) Coiled tail C) Big and amorphous head E) Bent tail F) Headless tail G) Bent neck H) Tail coiled around head and middle piece L) Free head M) Damaged head.

**Figure 4:**
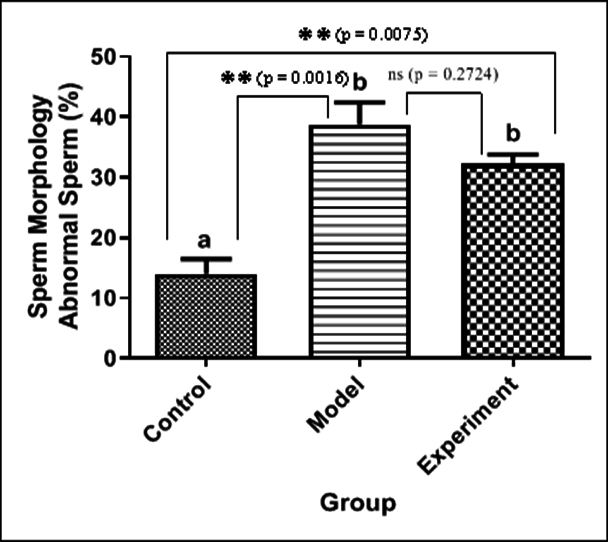
Sperm morphology / abnormal sperm diagram, **P < 0.01, ns: Not statistically significant.

Sperm morphology was examined using Diff-Quik staining. While the head shape was the main focus, the sperm tail (middle and basal segments) was also considered. Large or small heads, tapering, pyriform, round, shapeless, vacuolated ( > 2 vacuoles or more than 20% of the head is filled with unstained vacuole area), the presence of vacuoles in the post-acrosomal part, small or large acrosomal areas ( < 40% or > 70% of the head area), 2 heads, or any combination of these were considered as abnormal cases (Figure 3). The percentage of abnormal sperm cells is calculated and shown as a diagram (Figure 4). The average percentage of abnormal sperms in control rats was 14.01 ± 4.24%, whereas in cannabis-addicted rats, it was 38.77 ± 6.31%, and in GDNF-treated animals, it was 32.19 ± 2.79%. Despite GDNF treatment, the number of spermatozoa with abnormal morphology was still significantly higher than those of healthy control rats.

### DNA fragmentation

**Figure 5:**
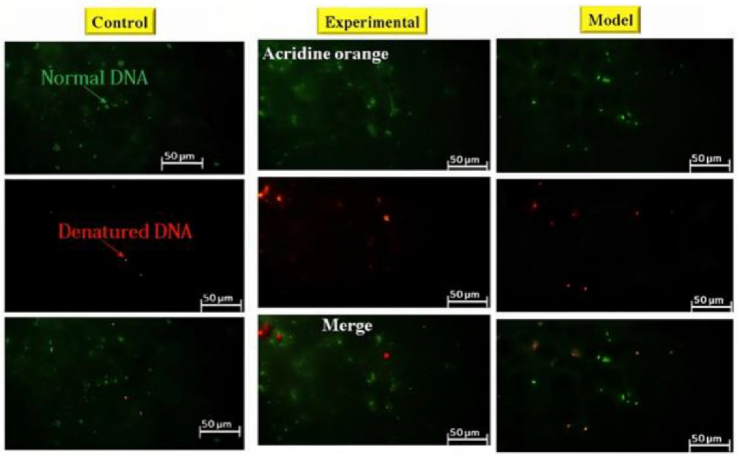
Evaluation of DNA damage in sperm cells using acridine orange staining.

Acridine orange staining was used to evaluate DNA damage in sperm cells (Figure 5). In this staining, normal DNA is seen in green and abnormal DNA in red. The analysis of sperm samples revealed a notable elevation in the proportion of denatured DNA among rats exhibiting addiction to cannabis. The administration of GDNF resulted in a decrease in the quantity of desaturated DNA compared to the addicted group. GDNF significantly alleviated the DNA damage caused by cannabis (Table IV).

### Sperm viability

**Figure 6:**
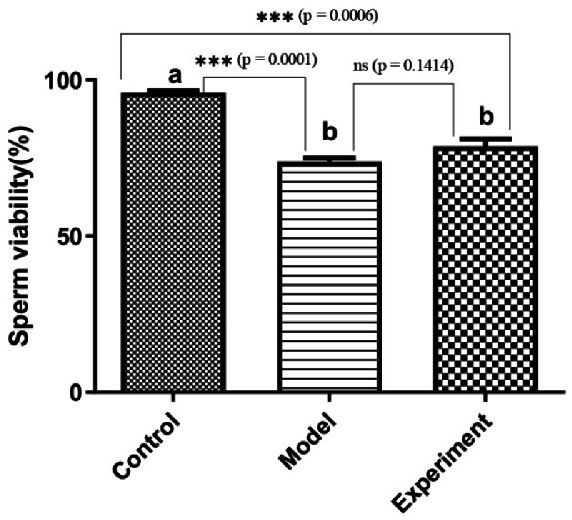
Analyze of sperm viability through trypan blue staining (***P < 0.001, ns: Not statistically significant).

Sperm viability was analyzed through trypan blue staining (Figure 6). The images obtained were used to calculate the percentage of viable and dead sperm in the groups of rats studied. The results revealed that the rate of viable sperm in cannabis-addicted rats was significantly lower than in the control group. Treatment with GDNF slightly increased the rate of viable sperm (p > 0.01).

**Table 1:** Comparison between groups in behavioral studies

**Dependent variable**		**Mean difference (I-J)**	**Std. Error**	**P-value**
**OFT**				
**Crossing**				
	**Control vs. Model**	17.667 ${ߐ}^{*}$	1.089	< 0.001***
	**Control vs. Experimental**	1.000	1.089	0.649 ${ߐ}^{\text{ns}}$
	**Model vs. Control**	-17.667 ${ߐ}^{*}$	1.089	< 0.001***
	**Model vs. Experimental**	-16.667 ${ߐ}^{*}$	1.089	< 0.001***
	**Experimental vs. Control**	-1.000	1.089	0.649 ${ߐ}^{\text{ns}}$
	**Experimental vs. Model**	16.667 ${ߐ}^{*}$	1.089	< 0.001***
**Risk behavior**				
	**Control vs. Model**	1.667 ${ߐ}^{*}$	0.385	0.012*
	**Control vs. Experimental**	1.333 ${ߐ}^{*}$	0.385	0.031*
	**Model vs. Control**	-1.667 ${ߐ}^{*}$	0.385	0.012*
	**Model vs. Experimental**	-0.333	0.385	0.679 ${ߐ}^{\text{ns}}$
	**Experimental vs. Control**	-1.333 ${ߐ}^{*}$	0.385	0.031*
**Grooming**				
	**Control vs. Model**	4.000 ${ߐ}^{*}$	0.816	0.006**
	**Control vs. Experimental**	6.000 ${ߐ}^{*}$	0.816	0.001**
	**Model vs. Control**	-4.000 ${ߐ}^{*}$	0.816	0.006**
	**Model vs. Experimental**	2.000	0.816	0.109 ${ߐ}^{\text{ns}}$
	**Experimental vs. Control**	-6.000 ${ߐ}^{*}$	0.816	0.001**
	**Experimental vs. Model**	-2.000	0.816	0.109 ${ߐ}^{\text{ns}}$
**Rearing**				
	**Control vs. Model**	16.000 ${ߐ}^{*}$	0.667	< 0.001***
	**Control vs. Experimental**	2.000	0.667	0.054 ${ߐ}^{\text{ns}}$
	**Model vs. Control**	-16.000 ${ߐ}^{*}$	0.667	< 0.001***
	**Model vs. Experimental**	-14.000 ${ߐ}^{*}$	0.667	< 0.001***
	**Experimental vs. Control**	-2.000	0.667	0.054 ${ߐ}^{\text{ns}}$
	**Experimental vs. Model**	14.000 ${ߐ}^{*}$	0.667	< 0.001***
**Sniffing**				
	**Control vs. Model**	7.000 ${ߐ}^{*}$	0.816	< 0.001***
	**Control vs. Experimental**	8.333 ${ߐ}^{*}$	0.816	< 0.001***
	**Model vs. Control**	-7.000 ${ߐ}^{*}$	0.816	< 0.001***
	**Model vs. Experimental**	1.333	0.816	0.304 ${ߐ}^{\text{ns}}$
	**Experimental vs. Control**	-8.333 ${ߐ}^{*}$	0.816	< 0.001***
	**Experimental vs. Model**	-1.333	0.816	0.304 ${ߐ}^{\text{ns}}$
**Napping**				
	**Control vs. Model**	-4.000 ${ߐ}^{*}$	0.471	< 0.001***
	**Control vs. Experimental**	0.000	0.471	1.000 ${ߐ}^{\text{ns}}$
	**Model vs. Control**	4.000 ${ߐ}^{*}$	0.471	0.001***
	**Model vs. Experimental**	4.000 ${ߐ}^{*}$	0.471	0.001***
	**Experimental vs. Control**	0.000	0.471	1.000 ${ߐ}^{\text{ns}}$
	**Experimental vs. Model**	-4.000 ${ߐ}^{*}$	0.471	0.001***
**Defecating**				
	**Control vs. Model**	4.000 ${ߐ}^{*}$	0.471	< 0.001***
	**Control vs. Experimental**	4.000 ${ߐ}^{*}$	0.471	< 0.001***
	**Model vs. Control**	-4.000 ${ߐ}^{*}$	0.471	< 0.001***
	**Model vs. Experimental**	0.000	0.471	1.000 ${ߐ}^{\text{ns}}$
	**Experimental vs. Control**	-4.000 ${ߐ}^{*}$	0.471	< 0.001***
	**Experimental vs. Model**	0.000	0.471	1.000 ${ߐ}^{\text{ns}}$
**SPT**				
**SDT**				
	**Control vs. Model**	37.333 ${ߐ}^{*}$	3.953	< 0.001***
	**Control vs. Experimental**	9.333	3.953	0.122 ${ߐ}^{\text{ns}}$
	**Model vs. Control**	-37.333 ${ߐ}^{*}$	3.953	< 0.001***
	**Model vs. Experimental**	-28.000 ${ߐ}^{*}$	3.953	0.001**
	**Experimental vs. Control**	-9.333	3.953	0.122 ${ߐ}^{\text{ns}}$
	**Experimental vs. Model**	28.000 ${ߐ}^{*}$	3.953	0.001**
**EPM**				
**Crossing**				
	**Control vs. Model**	8.000	2.893	0.043*
	**Control vs. Experimental**	1.667	2.893	0.837 ${ߐ}^{\text{ns}}$
	**Model vs. Control**	-8.000	2.893	0.033*
	**Model vs. Experimental**	-6.333	2.893	0.046*
	**Experimental vs. Control**	-1.667	2.893	0.837 ${ߐ}^{\text{ns}}$
	**Experimental vs. Model**	6.333	2.893	0.046*
**Risk behavior**				
	**Control vs. Model**	0.000	0.667	1.000 ${ߐ}^{\text{ns}}$
	**Control vs. Experimental**	0.667	0.667	0.603 ${ߐ}^{\text{ns}}$
	**Model vs. Control**	0.000	0.667	1.000 ${ߐ}^{\text{ns}}$
	**Model vs. Experimental**	0.667	0.667	0.603 ${ߐ}^{\text{ns}}$
	**Experimental vs. Control**	-0.667	0.667	0.603 ${ߐ}^{\text{ns}}$
	**Experimental vs. Model**	-0.667	0.667	0.603 ${ߐ}^{\text{ns}}$
**Rearing**				
	**Control vs. Model**	13.667 ${ߐ}^{*}$	0.720	< 0.001***
	**Control vs. Experimental**	7.000 ${ߐ}^{*}$	0.720	< 0.001***
	**Model vs. Control**	-13.667 ${ߐ}^{*}$	0.720	< 0.001***
	**Model vs. Experimental**	-6.667 ${ߐ}^{*}$	0.720	< 0.001***
	**Experimental vs. Control**	-7.000 ${ߐ}^{*}$	0.720	< 0.001***
	**Experimental vs. Model**	6.667 ${ߐ}^{*}$	0.720	< 0.001***
**Grooming**				
	**Control vs. Model**	2.667 ${ߐ}^{*}$	0.471	0.003**
	**Control vs. Experimental**	5.667 ${ߐ}^{*}$	0.471	< 0.001***
	**Model vs. Control**	-2.667 ${ߐ}^{*}$	0.471	0.003**
	**Model vs. Experimental**	3.000 ${ߐ}^{*}$	0.471	0.002**
	**Experimental vs. Control**	-5.667 ${ߐ}^{*}$	0.471	< 0.001***
	**Experimental vs. Model**	-3.000 ${ߐ}^{*}$	0.471	0.002**
**Sniffing**				
	**Control vs. Model**	1.333	0.609	0.151 ${ߐ}^{\text{ns}}$
	**Control vs. Experimental**	4.000 ${ߐ}^{*}$	0.609	0.001**
	**Model vs. Control**	-1.333	0.609	0.151 ${ߐ}^{\text{ns}}$
	**Model vs. Experimental**	2.667 ${ߐ}^{*}$	0.609	0.011*
	**Experimental vs. Control**	-4.000 ${ߐ}^{*}$	0.609	0.001**
	**Experimental vs. Model**	-2.667 ${ߐ}^{*}$	0.609	0.011*
**Napping**				
	**Control vs. Model**	-3.667 ${ߐ}^{*}$	0.272	< 0.001***
	**Control vs. Experimental**	0.000	0.272	1.000 ${ߐ}^{\text{ns}}$
	**Model vs. Control**	3.667 ${ߐ}^{*}$	0.272	< 0.001***
	**Model vs. Experimental**	3.667 ${ߐ}^{*}$	0.272	< 0.001***
	**Experimental vs. Control**	0.000	0.272	1.000 ${ߐ}^{\text{ns}}$
	**Experimental vs. Model**	-3.667 ${ߐ}^{*}$	0.272	< 0.001***
**Defecating**				
	**Control vs. Model**	3.333 ${ߐ}^{*}$	0.272	< 0.001***
	**Control vs. Experimental**	3.333 ${ߐ}^{*}$	0.272	< 0.001***
	**Model vs. Control**	-3.333 ${ߐ}^{*}$	0.272	< 0.001***
	**Model vs. Experimental**	0.000	0.272	1.000 ${ߐ}^{\text{ns}}$
	**Experimental vs. Control**	-3.333 ${ߐ}^{*}$	0.272	< 0.001***
	**Experimental vs. Model**	0.000	0.272	1.000 ${ߐ}^{\text{ns}}$
*The mean difference is significant at the 0.05 level. Data presented as Mean ± SD, ANOVA, and Tukey's test. *P < 0.05, **P < 0.01, ***P < 0.001, ns: Not statistically significant, OFT: Open-field test, SPT: Sucrose preference test, EPM: Elevated plus maze				

**Table 2:** Pathology H&E staining result

**Groups**		**Mean difference**	**q**	**P-value**	**95% CI of diff**
**Number of the spermatogonia/Tubule**					
	**Control vs. Experimental**	26.33	20.63	< 0.0001***	20.79 to 31.87
	**Control vs. Model**	27.67	21.67	< 0.0001***	22.13 to 33.21
	**Experimental vs. Model**	1.333	1.044	0.7511 ${ߐ}^{\text{ns}}$	-4.206 to 6.872
**Number of spermatocyte/Tubul**					
	**Control vs. Experimental**	43.67	10.16	0.0009***	25.01 to 62.32
	**Control vs. Model**	47.67	11.09	0.0006***	29.01 to 66.32
	**Experimental vs. Model**	4.000	0.9304	0.7950 ${ߐ}^{\text{ns}}$	-14.65 to 22.65
**Thickness of the Germ layer/(µm)**					
	**Control vs. Experimental**	69.99	7.043	0.0060**	26.87 to 113.1
	**Control vs. Model**	79.79	8.029	0.0031**	36.67 to 122.9
	**Experimental vs Model**	9.800	0.9862	0.7738 ${ߐ}^{\text{ns}}$	-33.32 to 52.92
**Number of Spermatid/Tubul**					
	**Control vs. Experimental**	276.4	19.45	< 0.0001***	214.7 to 338.0
	**Control vs. Model**	280.1	19.71	< 0.0001***	218.5 to 341.8
	**Experimental vs. Model**	3.718	0.2616	0.9814 ${ߐ}^{\text{ns}}$	-57.94 to 65.37
**Number of Sertoli/ Tubul**					
	**Control vs. Experimental**	25.33	18.08	< 0.0001***	19.25 to 31.41
	**Control vs. Model**	26.33	18.80	< 0.0001***	20.25 to 32.41
	**Experimental vs Model**	1.000	0.7137	0.8718 ${ߐ}^{\text{ns}}$	-5.079 to 7.079
**Number of Leydig cell/Tubul**					
	**Control vs. Experimental**	7.333	4.726	0.0358*	0.6010 to 14.07
	**Control vs. Model**	7.667	4.941	0.0299*	0.9344 to 14.40
	**Experimental vs. Model**	0.3333	0.2148	0.9874 ${ߐ}^{\text{ns}}$	-6.399 to 7.066
**Seminiferous basement membrane thickness/(μm)**					
	**Control vs. Experimental**	21.52	7.519	0.0043**	9.102 to 33.94
	**Control vs. Model**	21.95	7.668	0.0039**	9.528 to 34.36
	**Experimental vs. Model**	0.4260	0.1489	0.9939 ${ߐ}^{\text{ns}}$	-11.99 to 12.84
**Thickness of Internal diameter seminiferous (μm)**					
	**Control vs. Experimental**	-111.3	30.98	< 0.0001***	-126.9 to -95.69
	**Control vs. Model**	-115.8	32.25	< 0.0001***	-131.4 to -100.2
	**Experimental vs. Model**	-4.553	1.268	0.6620 ${ߐ}^{\text{ns}}$	-20.14 to 11.03
**Thickness of Outer diameter seminiferous/(µm)**					
	**Control vs. Experimental**	113.2	5.836	0.0145*	29.05 to 197.4
	**Control vs. Model**	109.8	5.661	0.016*	25.64 to 194.0
	**Experimental vs. Model**	-3.408	0.1757	0.9915) ${ߐ}^{\text{ns}}$	-87.59 to 80.78
Tukey's Multiple Comparison Test. Data presented as Mean ± SD, ANOVA, and Tukey test. The mean difference is significant at the 0.05 level. *P < 0.05, **P < 0.01, ***P < 0.001, ns: Not statistically significant					

**Table 3:** Sperm motility evaluated using the grading system (A-D)

**Groups**		**Mean difference**	**95.00% CI of diff.**	**P-value**
**Grade A**				
	**Control vs. Experiment**	5	-1.987 to 11.99	0.1505 ${ߐ}^{\text{ns}}$
	**Control vs. Model**	9.333	2.347 to 16.32	0.0150*
	**Experiment vs. Model**	4.333	-2.653 to 11.32	0.2180 ${ߐ}^{\text{ns}}$
**Grade B**				
	**Control vs. Experiment**	7.333	-3.197 to 17.86	0.1622 ${ߐ}^{\text{ns}}$
	**Control vs. Model**	14	3.470 to 24.53	0.0153*
	**Experiment vs. Model**	6.667	-3.863 to 17.20	0.2074 ${ߐ}^{\text{ns}}$
**Grade C**				
	**Control vs. Experiment**	4.667	-9.332 to 18.67	0.5907 ${ߐ}^{\text{ns}}$
	**Control vs. Model**	-11.33	-25.33 to 2.665	0.1042 ${ߐ}^{\text{ns}}$
	**Experiment vs. Model**	-16	-30.00 to -2.002	0.0295*
**Grade D**				
	**Control vs. Experiment**	-17	-24.65 to -9.346	0.0012**
	**Control vs. Model**	-12	-19.65 to -4.346	0.0071**
	**Experiment vs. Model**	5	-2.654 to 12.65	0.1919*
**Sperm Count (10^7^)**				
	**Control vs. Experiment**	8700000	5551499 to 11848501	0.0004***
	**Control vs. Model**	10213333	7064832 to 13361834	0.0001***
	**Experiment vs. Model**	1513333	-1635168 to 4661834	0.3660 ${ߐ}^{\text{ns}}$
Tukey's multiple comparison test. Data presented as Mean ± SD, ANOVA, and Tukey test. *P < 0.05, **P < 0.01, ***P < 0.001, ns: Not statistically significant				

**Table 4:** Sperm denaturation result

**Groups**	**Mean difference**	**P-value**	**95% CI of diff**
**Control vs. Experimental**	-14.67	0.0065**	-23.85 to -5.481
**Control vs. Model**	-25.67	0.0003***	-34.85 to -16.48
**Experimental vs. Model**	-11.00	0.0242*	-20.19 to -1.814
Tukey's multiple comparison test. Data presented as Mean ± SD, ANOVA. *P < 0.05, **P < 0.01, ***P < 0.001			

## 4. Discussion

In the present study, the impact of cannabis addiction on rat behavior and sperm parameters was examined, with a particular focus on the potential influence of GDNF in restoring these parameters. The findings indicated that rats had significant alterations in behavior as a result of cannabis addiction, with observable indications of despair and anxiety. In contrast, the administration of GDNF to rats with addiction resulted in a notable amelioration of these behavioral abnormalities. In the context of sperm parameters, it has been observed that cannabis addiction has a notable impact on various aspects, including morphology, motility, chromosomal integrity, and viability of sperms. The quantity of germ cells was notably decreased as a result of cannabis exposure. The empirical findings indicate that GDNF has shown a substantial capacity to ameliorate these alterations.

A notable global trend has been observed in recent years, indicating a decline in male fertility. There is a suggestion that exposure to chemicals may potentially play a role in the development of male reproductive problems. Environmental contaminants and pharmaceuticals can negatively impact male reproductive health. A significant increase has been observed in the use of cannabis for both medicinal and recreational purposes. Male reproductive health has been negatively impacted by exposure to phytocannabinoids (26). It has been demonstrated in previous studies that marijuana abuse leads to a decline in both the quantity and density of sperm in both human and animal subjects. It was shown that the disruption of spermatogenesis is the cause of this correlation. Previous research on rats has demonstrated the existence of cannabinoid receptors inside the somatic and germ cells of the testis. Recent studies have indicated that the activation of the GDNF pathway has the effect of diminishing both biochemical and behavioral alterations in rats that have been subjected to substance abuse (27).

To date, numerous animal and human research have been undertaken to investigate the impact of GDNF on alcohol misuse. The majority of these investigations consistently demonstrate the favorable influence of GDNF in mitigating alcohol consumption (28). Previous studies have demonstrated that administering a solitary dose of GDNF in rodents resulted in a prompt reduction in their self-administered alcohol consumption. The data that is now available suggests that the downregulation of GDNF is associated with the consequences generated by drug abuse. The precise way in which GDNF counters addiction is still unclear. It is worth mentioning that a growing body of research substantiates the efficacy of medications that emulate the impact of GDNF or enhance its synthesis as potentially potent and targeted therapeutic interventions for addiction. There has been a lack of research to investigate the impact of GDNF on cannabis addiction to date. According to the current study's findings, GDNF had a partial impact on spermatogenesis abnormalities caused by cannabis addiction, and it also reduces sperm DNA damage.

The discovery of GDNF has greatly enhanced the current knowledge of mammalian spermatogonial stem cells (SSCs). GDNF has been observed to be present in other organs, such as the ovary and testis, during the developmental stages. GDNF, a paracrine factor in the testis, is released by Sertoli cells postnatally. It has been identified as the sole factor responsible for the maintenance and self-renewal of SSCs in vivo and in vitro (29). Additional growth factors released by Sertoli cells, including basic fibroblast growth factor and epidermal growth factor, are crucial for the proliferation of SSCs in vitro. The necessity of their self-renewal of SSCs in vivo is still unclear. Significantly, the testis morphology of rats lacking GDNF appears to remain unchanged during prenatal development. Although rats that lack or have reduced levels of GDNF can reach adulthood and reproduce, examination of their testes at a microscopic level has revealed that the process of spermatogenesis is compromised. In the present investigation, the administration of exogenous GDNF exhibited the capacity to enhance DNA damage repair and enhance sperm motility in rats exhibiting addiction to cannabis.

In a study, the epigenetic mechanisms involved in controlling GDNF following the use of ethanol and subsequent withdrawal in a rat model were investigated. The mRNA expression in the N-acetylcysteine was substantially higher in the experimental group than in the control group. Within the experimental group, there was a notable decrease in GDNF mRNA expression levels within the VTA. Additionally, there was a substantial reduction in methylation levels within the noncoding regulatory element in both the VTA and N-acetylcysteine as compared to the control group. Alterations in the GDNF mRNA expression within the brain region were observed to match changes in the DNA methylation patterns of the GDNF promoter in a rat model (30).

It was demonstrated that GDNF is effective in reducing the negative effects of substance misuse. Indeed, their research has shown that the reduction of endogenous GDNF levels or the inhibition of the GDNF pathway leads to an elevation in several biochemical markers and behavioral adjustments in individuals exposed to substances such as psychostimulants, opioids, and ethanol. It is not clear how GDNF exerts its effects on addiction. GDNF has both immediate and long-term effects on dopaminergic neurons. GDNF's influence on mitogen-activated protein kinases is responsible for the immediate effect, while changes in tyrosine hydroxylase level are the long-term effect. These potential contributors are considered to have an overall effect on GDNF. The effects induced by GDNF have the potential to result in synaptic remodeling, as well as alterations in the reactivity of the mesolimbic dopaminergic system, which is involved in regulating motivation and reward processing (13). The mechanism of action of GDNF was not explored in this study; however, our findings indicate that the administration of this molecule to rats with cannabis addiction can mitigate sperm DNA damage and enhance sperm motility.

## 5. Conclusion

This study evaluated the therapeutic effects of GDNF on addiction-related behaviors and spermatogenesis in cannabis-addicted rats. The findings revealed that GDNF significantly reduced addiction-related behaviors and improved spermatogenesis in this animal model. These results provide valuable evidence supporting the therapeutic potential of GDNF in managing cannabis use disorders and improving fertility outcomes. Further research is warranted to investigate the underlying mechanisms and validate these effects in human models.

##  Data Availability

All the data obtained from the research are mentioned in this article.

##  Author Contributions

M. Heydari Nasrabadi and R. Laleh designed the study and conducted the research. All authors monitored, evaluated, and analyzed the results of the study. R. Laleh wrote the manuscript. Further, M. Heydari Nasrabadi reviewed the article. All authors approved the final manuscript and take responsibility for the integrity of the data.

##  Conflict of Interest

All authors declare that there is no conflict of interest.
